# Hepatobiliary and pancreatic hemorrhage: Technical tools and tricks

**DOI:** 10.1016/j.sopen.2021.10.001

**Published:** 2021-10-14

**Authors:** Chad G. Ball

**Affiliations:** Foothills Medical Centre, 1403 29 St NW, Calgary, Alberta, T2N 2T9

## Abstract

Ongoing hemorrhage from hepatobiliary and pancreatic injuries continues to daunt even the most experienced surgeon. Despite the widespread centralization of elective hepatopancreatobiliary (HPB) surgery to high-volume centers, HPB trauma remains relatively common and requires a rapid and thoughtful approach [[1], [2], [3], [4], [5], [6], [7], [8], [9], [10], [11], [12], [13]].

## PANCREAS INJURIES

Although blood flow within the pancreatic gland is impressive, the dominant source of hemorrhage associated with pancreatic trauma remains the mesenteric venous structures that surround it. More specifically, the superior mesenteric, portal, inferior mesenteric, and splenic veins remain the prime sources. Although the anatomy of the portal and superior mesenteric veins is relatively constant, the insertion point of the inferior mesenteric vein can vary dramatically (ie, insertion into the splenic and/or portal veins). It is also generally stated that the portal vein does not possess any branches arising from its anterior surface (ie, immediately posterior to the pancreatic neck). As with many dogmatic anatomic comments, this "rule" is frequently broken. The presence of large venous tributaries from the portal vein into the head and uncinate of the pancreas is absolute however. The largest of these are the gastroepiploic trunk and the first jejunal venous branch. Hemorrhage from these structures can be torrential and unforgiving.

Although portal venous injuries in the retropancreatic location are notoriously difficult to access, human anatomy has provided us with a powerful temporizing maneuver. More specifically, digital pressure from the front of the gland is often adequate to temporarily control venous hemorrhage via simple compression. In cases where this fails, a rapid Kocher maneuver is required to allow concurrent anterior and posterior digital pressure and therefore occlusion of the portal vein and distal SMV. This maneuver provides the surgeon time to add a second suction device, call for experienced assistance, notify the anesthesiologist for predicted massive blood loss, and have the nursing staff ready all vascular instrumentation and suture selections that the surgeon will require. If the hemorrhage appears to arise from the bottom of the pancreas (ie, uncinate/head), it is helpful to rapidly mobilize the right colon to provide improved inferior exposure and eventually control. Inspection of the right lateral small bowel mesentery will also provide insight into possible hemorrhage caudal to the pancreatic head/uncinate. As with most massive bleeding, the importance of an educated assistant who can expose the venous injury (both suctioning of blood and retraction of adjacent organs) cannot be overstated. Excellent help is typically the difference between completing an efficient and smooth repair compared to flailing with massive blood loss and patient demise.

If pressure and packing do not persistently control hemorrhage from the retropancreatic portion of the portal vein (a very unusual circumstance), then rapid exposure and subsequent ligation/repair of the vessel may be required. This can be achieved by dividing the neck/body of the pancreas gland (ie, to allow direct visualization of the vessel). This maneuver is discussed significantly more often in the literature than it is actually performed in the real world. It also carries with it a substantial risk of inadvertently enlarging the venous injury (given near ubiquitous rough dissection in a poorly visualized field). If this maneuver is triggered however, rapidly place 4 retraction sutures (3-0 Prolene on MH needles) through the pancreatic neck in a figure-of-eight manner immediately lateral and medial to the portal vein (at both the top and bottom of the gland). There is significant risk of ligating the hepatic artery at the top, so you must be extremely accurate. This is not the time to flail or lose focus. These 4 sutures will provide significant retraction from both sides of the pancreatic neck, allowing the surgeon to use high-voltage Bovie electrocautery to transect the pancreas quickly (in combination with adequate ongoing suction). Remember that the vein is not typically dissected off of the back of the pancreas (ie, elective pancreatoduodenectomy), so you need to slow down as you move closer to the posterior margin of the pancreas. Repairs to the portal or superior mesenteric veins themselves are generally performed with a 5-0 or 6-0 RB-1 Prolene (once control is obtained with proximal and distal vascular clamps or digital pressure and exposure by an experienced colleague).

When necessary, venous tributaries from the portal vein can generally be ligated. Even the portal vein itself can be ligated in a damage control scenario. Interestingly, these patients display a superior survival when evaluating the literature as a whole (versus those who undergo portal and superior mesenteric venous repairs). This likely reflects the varying comfort level among surgeons attempting to address these difficult injuries. Another possible solution is to place a temporary intravascular shunt (TIVS) in continuity for major portal venous injuries. Portal veins are best shunted with a 22F to 26F chest tube, or large nasogastric feeding tube for small women. Once inserted into the vessel (in-line), the TIVS can be locked into place via silk ties or double vessel loops that are tightened/locked with clips. If silk ties are selected (generally more secure for venous structures), it must be remembered that the vessel itself will need to be trimmed back proximal to the silk to ensure there is no ischemia at the time of the reconstruction. This may become a problem in areas where every bit of vessel length is critical (ie, necessitating a graft at the time of reconstruction).

Ongoing hemorrhage from the splenic vein is much less treacherous. Bleeding to the anatomic left of the portal vein can be solved via a rapid distal pancreatectomy/splenectomy with bulk ligation of the splenic artery and vein. Energy instrumentation and staplers can make this endeavor simple and efficient. More specifically, divide the short gastrics and gastroepiploics, mobilize the transverse and left colon, and finally free the spleen with an energy instrument that works well within pools of blood (LigaSure Impact, Covidien). A multitude of staplers can then be effectively utilized to divide the pancreatic body concurrent to ligation of the splenic artery and vein. The TX-30v linear stapler (Ethicon) is a workhorse for the elective HPB surgeon and is superb for this indication. Alternatively, in the context of a soft gland, a laparoscopic stapler (60 mm length vascular load, 2.5 mm staple height) can also be used to divide these structures en masse. The dominant risk is mistaking the hepatic artery for the proximal splenic artery and dividing it. A quick test clamp of the splenic artery with a large bulldog clamp (to ensure a normal persistent pulse within the porta hepatis) eliminates this potential disaster. Prior to placing either stapler, however, the surgeon must rapidly dissect around the pancreatic body and place a vessel loop or umbilical tape for complete control of the gland. This is often best done with a single well-educated finger. Remember that the retroperitoneum at this location (ie, aorta is deep to this site) is generally spared from hemorrhage and easily accessible from the bottom once the transverse colon is mobilized caudally by approximately 1 cm. The splenic vein will remain stuck to the underside of the elevated pancreas, and the splenic artery can be palpated. Although this short series of maneuvers may sound challenging, it becomes much easier when rapidity is in demand. The most extreme damage control maneuver for a splenic venous injury remains bulk ligation with a large suture, followed by packing.

Although it is beyond the aims of this article, the dominant postoperative complications surrounding pancreatic (and duodenal) injuries remain leaks from preceding pancreatoduodenal closures and/or anastomoses. Critically injured patients rarely tolerate the physiologic consequences of uncontrolled leaks. Pancreatic juices are also highly dangerous in the context of a fresh vascular repair, anastomosis, or TIVS. As a result, generous closed suction drainage must be considered to control any potential pancreatic leaks after the ongoing hemorrhage has been stopped.

## LIVER INJURIES

The dominant challenge with hepatic trauma generally surrounds the management of the hemodynamically unstable patient with a bleeding, high-grade liver injury. More specifically, these injuries can be difficult to expose, temporize, and/or repair for any surgeon who does not make his or her living in this region of the upper abdomen. These patients often present in physiologic extremis and therefore require damage control resuscitation techniques. Early recognition of their critical condition, as well as immediate hemorrhage control, is essential. Unlike the spleen and kidney, the liver cannot generally be resected in a rapid on-demand basis. Regardless of your training, these injuries will engage all of your senses, test your technical skills, require the utmost focus, and demand great teamwork from you and your colleagues.

Patients with major injury as a result of either blunt or right upper quadrant penetrating trauma must undergo an immediate Extended Focused Assessment with Sonography for Trauma examination in the trauma bay to confirm the presence of large-volume intraperitoneal fluid. This examination is repeatable and should be used to reevaluate patients in urban centers who present immediately following their injuries. Massive transfusion protocols as part of a damage control resuscitation must be initiated early during the patient assessment process. If the patient rapidly stabilizes their hemodynamics, they should undergo an emergency computed tomography (CT) scan of their torso. If they remain clinically unstable, they must be transferred to the operating theater without delay. Hemorrhage control is the dominant driver limiting survival. Collateral issues such as optimal intravenous access, imaging of other areas (brain, spine, bones), and fracture fixation are secondary problems.

Thankfully, not all patients with liver injuries are actively dying secondary to hemorrhage. More specifically, in hemodynamically stable patients without CT evidence of a hepatic arterial blush, admission and close observation are warranted. In hemodynamically stable patients with a hepatic arterial blush, immediate transfer to the interventional angiography suite (or hybrid operating room) is recommended. Hepatic angiography and/or portography with selective embolization is indicated with either autologous clot or absorbable embolization medium. In persistently hemodynamically unstable patients, however, an immediate laparotomy is essential. More to the point, early recognition of a patient with ongoing hepatic hemorrhage and immediate transfer to the operating theater are crucial. Delays will lead to the loss of life.

The patient should be rapidly prepared and draped with available access from the neck to the knees. Vascular instruments and balloons must be open and at the ready. A midline laparotomy from xiphoid process to pubic bone should be performed with 3 passes of a sharp scalpel. The peritoneal cavity should be packed in its entirety with laparotomy sponges for patients with blunt liver injuries. Although the ligamentum teres can be ligated, the falciform ligament may be left intact (especially in blunt trauma to the right lobe of the liver). This offers a medial wall against which to improve packing pressure. The right upper quadrant should be evaluated prior to any potential intraperitoneal packing for penetrating injuries. If hemorrhage continues, an early Pringle maneuver (clamping of the porta hepatis with a vascular clamp) is recommended. This is both diagnostic and potentially therapeutic. If bleeding continues despite application of a Pringle clamp, a retrohepatic inferior vena cava (IVC) or hepatic venous injury is likely (assuming that a replaced left hepatic artery is not the source of inflow occlusion failure). Critically injured patients in physiologic extremis do not tolerate extended Pringle maneuvers to the same extent as patients with hepatic tumors undergoing elective hepatic resection. Forty minutes represents the upper limit of viable. If the liver hemorrhage responds to packing but continues to hemorrhage when unpacking is completed, they should be repacked and transferred to the ICU with an open abdomen once damage control of concurrent injuries is complete. Cover the liver with a plastic layer of sterile x-ray cassette material to avoid capsular trauma upon eventual unpacking. It should be reemphasized that all damage control procedures should be completed in less than 1 hour. Return to the operating suite in patients with packed abdomens should occur in 48–72 hours (assuming hypothermia, coagulopathy, and acidosis are corrected).

If the liver hemorrhage control is dependent on maintenance of a Pringle maneuver despite packing, call for senior assistance, mobilize the right lobe, and suture the IVC or hepatic veins with 4-0 Prolene on SH needles. These patients may also require total vascular exclusion/occlusion (TVE) of the liver. This technique involves complete occlusion of the infrahepatic IVC, suprahepatic IVC, porta hepatis (Pringle maneuver), as well as an aortic cross-clamp within the abdomen. If TVE is pursued without concurrent clamping of the aorta, the patient will often arrest due to a lack of coronary perfusion. Prior to performing TVE of the liver, it is imperative to allow the anesthetic team to resuscitate the patient to the best of their ability to facilitate IVC clamping. We prefer to obtain suprahepatic IVC control within the abdomen in patients with a normal length of IVC inferior to the diaphragm. An alternate approach involves accessing the IVC immediately prior to its entry into the heart (ie, within the pericardial sac). This 2-cm length of IVC is easily accessible by opening the pericardial sac following division of the central tendon of the diaphragm. Alternatively, it can also be accessed from the thorax if a thoracotomy has already been performed. Control of the infrahepatic IVC can be rapidly gained by opening the overlying peritoneum and bluntly encircling the IVC cephalad to the right real vein (ie, no lumbar veins reside above the renal veins).

Veno-veno bypass is also a theoretical option in some very specific scenarios but is rarely required if the patient can be adequately resuscitated to allow for IVC clamping. Furthermore, a lack of transplantation training in most trauma/general surgeons precludes expeditious use of this bypass.

In the case of central hepatic gunshot wounds or deep central lacerations where access and exposure are difficult, ongoing hemorrhage should be stopped with balloon occlusion. Either a Blakemore esophageal balloon or variant (red rubber catheter with overlying Penrose drain and 2 silk occlusion ties) is exceptional at stopping ongoing bleeding at the bottom of deep central hepatic injury tracts (including retrohepatic IVC injuries). Foley catheters of varying sizes are also helpful. These should be deflated approximately 72 hours after the initial placement. If hemorrhage continues, they should be reinflated and left in vivo for 3 additional days.

Another excellent damage control option for major IVC disruption, portal vein injuries, and combined portal venous/hepatic arterial trauma is the use of a TIVS. Although a large variety of tubes can be utilized as a TIVS [chest tubes (adult and pediatric), nasogastric tubes, carotid shunts, pediatric feeding tubes], they do not need to be heparin bonded. More specifically, TIVS typically fails for 1 of 3 reasons: (1) selection of a tube that is too small for the caliber of the disrupted vessel, (2) kinking of the tube itself, and (3) inadequate concurrent outflow (eg, shunting of an iliac artery without ensuring adequate iliac venous outflow). IVC injuries in adults are usually best approximated with a 32F to 36F chest tube. Portal veins are best shunted with a 22F to 26F chest tube, or large nasogastric feeding tube for small women. Hepatic arteries are best served by inserting pediatric nasograstric or feeding tubes. These TIVS may be locked into place with either silk ties or double-looped vessel loops and locking clips. As previously mentioned, the surgeon should consider the latter method in scenarios where maximizing the vessel distance is critical because the vessel will need to be further trimmed back beyond the silk ties when reconstruction is eventually attempted.

Vascular reconstruction following insertion of a TIVS should ideally involve an experienced HPB surgeon. The timing of this reconstruction will depend entirely upon the physiological and biochemical recovery of the patient. As soon as this is achieved in the critical care suite, the patient should return to the operating theater for repair. The surgeon must also ensure that a wide range of potential conduits is available and ready (saphenous vein, bovine pericardium, ringed and nonringed synthetic). One superb conduit choice for IVC reconstruction following TIVS removal is bovine pericardium (or biologic mesh) that is fashioned into a tube of the appropriate size (usually wrapped around a bulb syringe with a single firing of a laparoscopic stapler to convert a sheet into a tube). This conduit performs quite well in leaking/infected traumatic fields.

Although TIVS has revolutionized damage control trauma scenarios, the traditional damage control option for vascular trauma of ligation remains relevant. It is clear based on a literature review of portal venous and superior mesenteric venous trauma that ligation of this vessel, rather than reconstruction, is often superior. This observation is likely multifactorial but almost certainly relates to surgeon unfamiliarity (ie, time required to expose, control, and repair) with these vessels in anatomically hostile regions. Similarly, ligation of the IVC is also well recognized as a successful damage control maneuver. If the IVC is ligated, wrapping a patient's legs with compression garments, elevation of patient's lower extremities above the heart, and judicious fluid management for 5 postoperative days are critical.

Although unusual, patients with penetrating injuries to the hepatic artery will present as critically ill and may require ligation (assuming the portal vein is intact). Portal vein injuries should ideally be repaired with 5-0 or 6-0 Prolene once control is obtained. Clamps above and below the injury are essential for visualization. Alternate damage control options include TIVS with a small chest tube conduit or ligation (assuming the hepatic artery is intact).

If an atrial–caval shunt is contemplated, 2 experienced surgical teams (1 for the chest and 1 for the abdomen) are essential to ensure both rapidity and efficiency. The decision to pursue this shunt must be made early in the exploration process. Unfortunately, these shunts rarely result in patient salvage in even the most experienced trauma centers. If a center and/or surgical team considers this maneuver to be part of their armamentarium for treating ongoing hemorrhage from retrohepatic injuries, a prestocked kit with all the necessary items must be readily available. Similar to utilizing TIVS and occlusion balloons, demanding these instruments in the wee hours of the morning among a stressed clinical team for a decompensating patient is likely to fail. Remember that Allis clamps are also excellent for the initial control of most venous hemorrhage.

## SUMMARY

In conclusion, massive ongoing hemorrhage associated with pancreatic trauma is typically compressible with a well-educated hand/finger. A detailed knowledge of anatomy and a talented assistant will make the difference between a huge save and a long presentation at morbidity and mortality conference.

Although the published history of hepatic trauma is littered with descriptions of technical maneuvers ordered in a hierarchical scheme, very few are relevant in the context of modern trauma care. Packing of hepatic hemorrhage controls the vast majority of ongoing bleeding in critically ill patients. Selective use of vessel ligation, parenchyma resection, and hepatic transplant remain less common strategies. Ongoing hemorrhage from major hepatic injuries remains the most challenging of all intraperitoneal injuries due to issues with exposure, blood flow, and difficult technical repairs. Initiate damage control resuscitation and massive transfusion protocols early in your assessment. Rapid completion of damage control procedures is essential (< 1 hour). Flailing and indecision lead to prolonged operative times and patient demise. If diagnosis and therapy are rapid, patients who present in physiologic extremis as a result of major hepatic hemorrhage have a good chance of survival in the context of a prolonged hospital stay. Elective liver surgeons can be of superb assistance when available.

## Author Contribution

Dr. Ball wrote and edited all components of this manuscript.

## Conflict of Interest

Dr. Ball has no conflicts of interest to declare.

## Funding Source

There was no funding for this article.

## Ethics Approval

No patient data were included in this manuscript. No ethics approval was obtained.

## References

[1] Moore E.E., Cogbill T.H., Malangoni M.A., Jurkovich G.J., Champion H.R., Gennarelli T.A. (1990). Organ injury scaling, II: pancreas, duodenum, small bowel, colon, and rectum. J Trauma.

[2] Biffl W.L., Zhao F.Z., Morse B., McNutt M., Lees J., Byerly S. (2021). A multicenter trial of current trends in the diagnosis and management of high-grade pancreatic injuries. J Trauma Acute Care Surg.

[3] Joos E., de Jong N., Ball C.G., Quigley S., Trottier V., Masse M. (2021). Time to operating room matters in modern management of pancreatic injuries: a national review of the management of adult pancreatic injury at level 1 trauma centers. J Trauma Acute Care Surg.

[4] Biffl W.L., Moore E.E., Croce M., Davis J.W., Coimbra R., Karmy-Jones R. (2013). Western Trauma Association critical decisions in trauma: management of pancreatic injuries. J Trauma Acute Care Surg.

[5] Ball C.G., Clements T.S., Kirkpatrick A.W., Vogt K., Biffl W., Hameed S.M. (2021). Inviting a friend to evaluate potential grade III pancreatic injuries: are they truly occult, or simply missed on CT?. J Trauma Acute Care Surg.

[6] Ball C.G., Biffl W.L., Vogt K., Hameed S.M., Parry N.G., Kirkpatrick A.W. (2021). Does drainage or resection predict subsequent interventions and long-term quality of life in patients with grade IV pancreatic injuries: a population-based analysis. J Trauma Acute Care Surg.

[7] Lucas C.E., Ledgerwood A.M. (1976). Prospective evaluation of hemostatic techniques for liver injuries. J Trauma.

[8] Feliciano D.V., Mattox K.L., Jordan G.L. (1981). Intra-abdominal packing for control of hepatic hemorrhage: a reappraisal. J Trauma.

[9] Ball C.G., Wyrzykowski A.D., Nicholas J.M., Rozycki G.S., Feliciano D.V. (2011). A decade’s experience with balloon tamponade for the emergency control of hemorrhage. J Trauma.

[10] Kozar R.A., Feliciano D.V., Moore E.E., Cocanour C.S., West M.A., Davis J.W. (2011). Western Trauma Association/critical decisions in trauma: operative management of adult blunt hepatic trauma. J Trauma.

[11] Pachter H.L., Feliciano D.V. (1996). Complex hepatic injuries. Surg Clin North Am.

[12] Rozycki G.S., Ochsner M.G., Feliciano D.V., Thomas B., Boulanger B.R., David F.E. (1998). Early detection of hemoperitoneum by ultrasound investigation of the right upper quandrant: a multicenter study. J Trauma.

[13] Pachter H.L. (2012). Prometheus bound: evolution in the management of hepatic trauma—from myth to reality. J Trauma.

